# Supplementary material for: Virgin and UV-weathered polyamide microplastics posed no effect on the survival and reproduction of Daphnia magna
Source: PeerJ. 2022 May 30;10:e13533. doi: 10.7717/peerj.13533 (PMC9161812; doi:10.7717/peerj.13533)
Supplement: Supplemental Information 1 [file peerj-10-13533-s001.docx]

Supplemental file

**Do virgin and UV-weathered polyamide microplastics affect *Daphnia magna* reproduction?**

Alla Khosrovyan ^1^ *, Anne Kahru ^1, 2^

^1 National Institute of Chemical Physics and Biophysics, Laboratory of Environmental Toxicology, 23 Akadeemia tee, 12618, Tallinn, Estonia. Email:^ [^alla.khosrovyan@kbfi.ee^](mailto:alla.khosrovyan@kbfi.ee)

1. ^Estonian Academy of Sciences, 6 Kohtu, 10130, Tallinn, Estonia. Email:^ [^anne.kahru@kbfi.ee^](mailto:anne.kahru@kbfi.ee)

Images of polyamide particle before (virgin) and after UV-C irradiation for 26 days (UV-weathered) were taken with the help of the Zeiss Confocal laser scanning microscope LSM 800. Objective Plan-Apochromat 20x/0.8 M27. Laser wavelength 404 nm, detection wavelength 400-531 nm).

In total, 40 images of particle surfaces were scanned (20 for virgin plastics and 20 for UV-weathered ones).


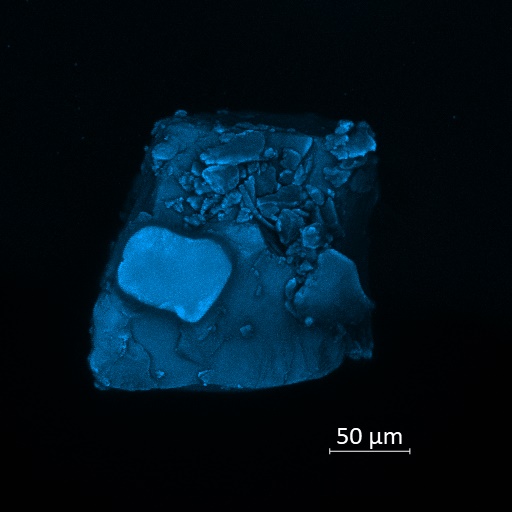

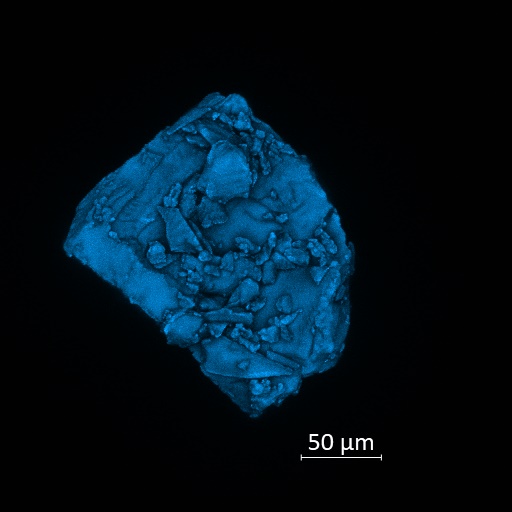

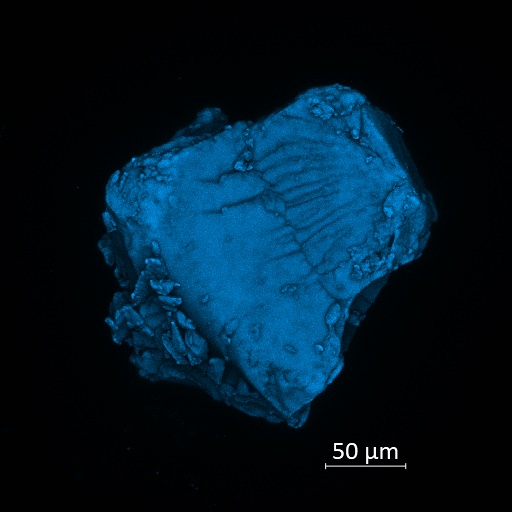

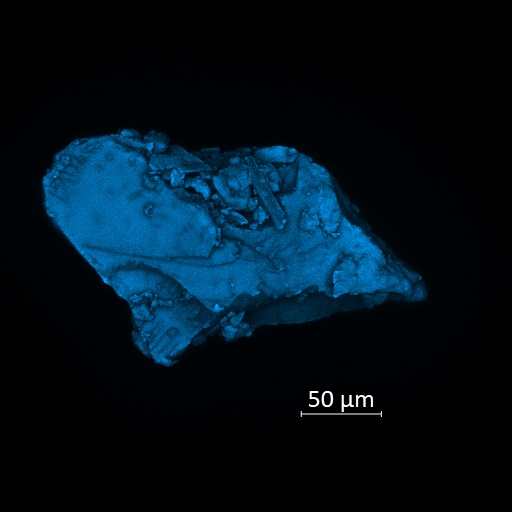


**1**

**2**

**3**

**4**

**5**


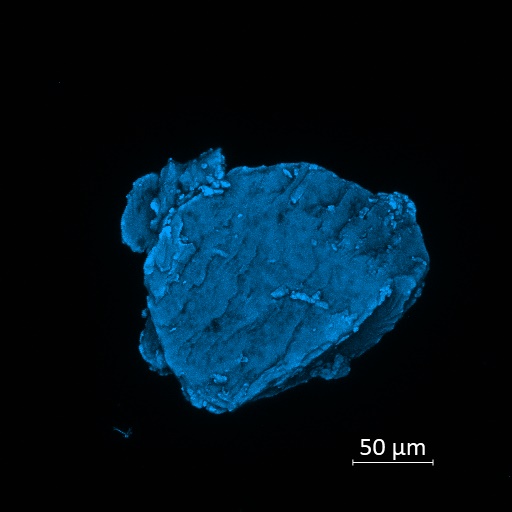

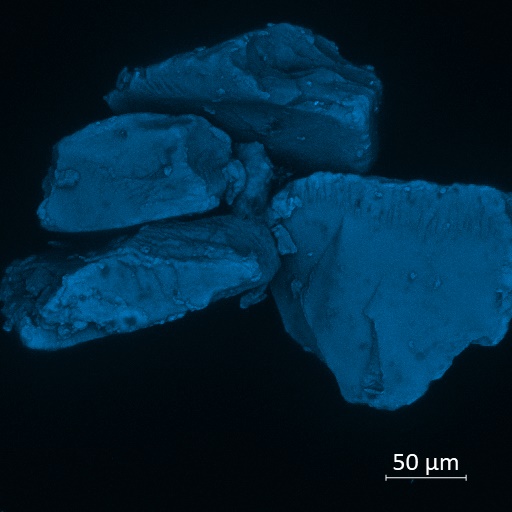

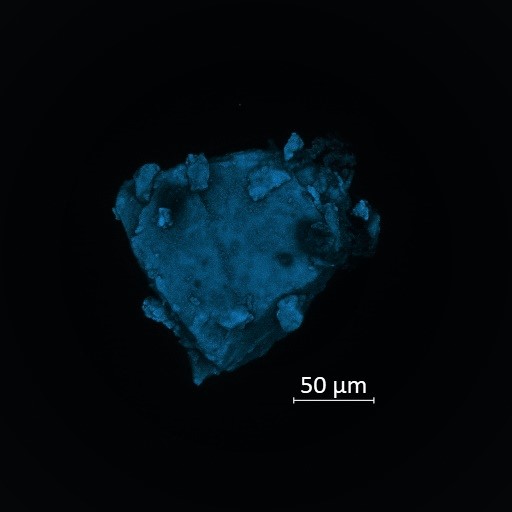

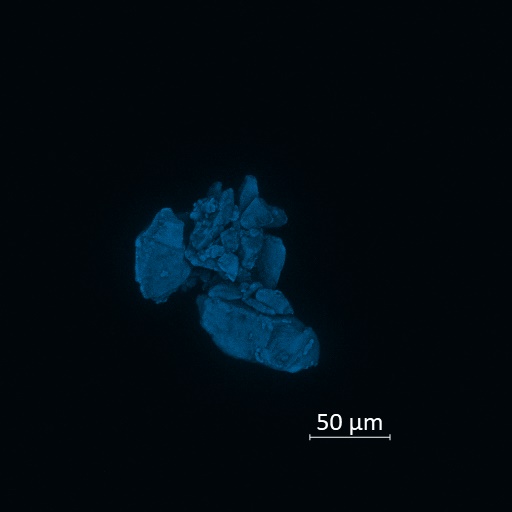


**5**

**6**

**7**

**8**

Fig. S1. Selected images of polyamide particle surface before (virgin) and after UV-C irradiation for 26 days (UV-weathered). Virgin (images 1-4), UV-weathered (images 5-8).

Table S1. Physico-chemical parameters of water from Lake Ülemiste (Estonia)

| pH | Conductivity µS cm^−1^ | Oxygen | NO_3_^-^ | NO_2_^−^ | NH_4_^+^ mg L^−1^ | PO_4_^3−^ | TOC | TP | TN |
| --- | --- | --- | --- | --- | --- | --- | --- | --- | --- |
|  |  |  |  |  |  |  |  |  |  |
| 8.55 | 393 | 10.9 | <1 | <0.003 | 0.007 | <0.02 | 11.2 | 0.032 | 1.1 |

^TOC: total organic carbon; TP: total phosphorus; TN: total nitrogen.^

Table S2. Details of the test of difference from control performed by the one-way ANOVA in R (version 4.1.3 (2022-03-10)): degrees of freedom (DF, residuals), test statistic (F value from the F test), the exact p-value (P), and effect size (Eta^2^). Test: OECD 211 *Daphnia magna* reproduction test. Significance: p>0.05.

|  | **Treatment:**  **virgin (PA-MP) and UV-weathered (UV-PA-MP) polyamide spiked to the natural water (Lake Ülemiste, Estonia)** | | | |
| --- | --- | --- | --- | --- |
|  |  |  |  |  |
|  | **DF** | **F value** | **P** | **Eta^2^** |
| **1^st^ experiment** |  |  |  |  |
|  |  |  |  |  |
| **UV-PA-MP** |  |  |  |  |
| 100 mg L^-1^ | 17 | 0.068 | 0.798 | 0.0039 |
| 300 mg L^-1^ | 17 | 0.034 | 0.857 | 0.0019 |
|  |  |  |  |  |
| **PA-MP** |  |  |  |  |
| 100 mg L^-1^ | 17 | 0.176 | 0.68 | 0.01 |
| 300 mg L^-1^ | 16 | 0.23 | 0.638 | 0.014 |
|  |  |  |  |  |
| **2^nd^ experiment** |  |  |  |  |
|  |  |  |  |  |
| **UV-PA-MP** |  |  |  |  |
| 100 mg L^-1^ | 18 | 0.128 | 0.725 | 0.007 |
| 300 mg L^-1^ | 18 | 0.001 | 0.98 | 3.5E-05 |
|  |  |  |  |  |
| **PA-MP** |  |  |  |  |
| 100 mg L^-1^ | 18 | 0.038 | 0.848 | 0.002 |
| 300 mg L^-1^ | 18 | 0.039 | 0.847 | 0.002 |
|  |  |  |  |  |
|  | **Treatment:**  **UV-weathered (UV-PA-MP) polyamide and/or surfactant Tween 20 spiked to the natural water (Lake Ülemiste, Estonia)** | | | |
|  |  |  |  |  |
| **1^st^ experiment** |  |  |  |  |
|  |  |  |  |  |
| **UV-PA-MP** |  |  |  |  |
| 300 mg L^-1^ |  |  |  |  |
| **Tween 20** |  |  |  |  |
| 7 mg L^-1^ | 18 | 2.217 | 0.154 | 0.11 |
|  |  |  |  |  |
| **Tween 20** |  |  |  |  |
| 7 mg L^-1^ | 18 | 0.08 | 0.781 | 0.0044 |
|  |  |  |  |  |
| **2^nd^ experiment** |  |  |  |  |
|  |  |  |  |  |
| **UV-PA-MP** |  |  |  |  |
| 300 mg L^-1^ |  |  |  |  |
| **Tween 20** |  |  |  |  |
| 7 mg L^-1^ | 18 | 0.001 | 0.981 | 3.2E-05 |
|  |  |  |  |  |
| **Tween 20** |  |  |  |  |
| 7 mg L^-1^ | 18 | 0.91 | 0.353 | 0.048 |
